# Fiber Distribution and Myelination of Dopaminergic Neurons in the Medial Forebrain Bundle of a Rodent Depression Model

**DOI:** 10.1002/jnr.70093

**Published:** 2025-11-16

**Authors:** Zhuo Duan, Yixin Tong, Xiongpeng Weng, Volker A. Coenen, Máté D. Döbrössy

**Affiliations:** ^1^ Department of Stereotactic and Functional Neurosurgery, Laboratory of Stereotaxy and Interventional Neurosciences University Freiburg Medical Center Freiburg im Breisgau Germany; ^2^ Department of Stereotactic and Functional Neurosurgery University Freiburg Medical Center Freiburg im Breisgau Germany; ^3^ Faculty of Medicine University of Freiburg Freiburg im Breisgau Germany; ^4^ Faculty of Biology University of Freiburg Freiburg im Breisgau Germany

**Keywords:** DBS, dopaminergic fibers, medial forebrain bundle, myelination, rodent depression model

## Abstract

It has been hypothesized that the rapid/long‐lasting antidepressant action observed following medial forebrain bundle DBS in clinical trials of Treatment‐Resistant Depression patients could partially be driven by modulation of the midbrain‐to‐forebrain dopaminergic fibers. The study investigated the spatial distribution and myelination status of dopaminergic fibers within the rodent equivalent structure in a preclinical depression model, the Flinders Sensitive Line (FSL). Fixed, sliced brain sections were double stained with anti‐tyrosine hydroxylase/anti‐dopamine‐β‐hydroxylase antibodies to distinguish dopaminergic from noradrenergic fibers, and with anti‐tyrosine hydroxylase/anti‐myelin antibodies to specifically investigate myelination. Quantification was done at six predefined segments. The dopaminergic fibers coursing through the medial forebrain bundle were small diameter, unmyelinated and mainly restricted to the dorsolateral quadrant on the AP axis. Analyses across six predefined planes revealed significantly fewer dopaminergic fibers in FSL males (vs control males, and vs. FSL females) at AP −2.8 mm, the segment corresponding to the medial forebrain bundle DBS target in the rat. No differences were observed elsewhere along the medial forebrain bundle. Previously reported differences in stimulation‐evoked dopamine release in the FSLs could be due to differences in the numbers of recruited dopaminergic fibers.

## Graphical abstract

The study investigated the spatial distribution and myelination status of dopaminergic fibers coursing through the medial forebrain bundle (mfb) in a rodent depression model (Flinders Sensitive Line, FSL) and Control animals, in males and females. All dopaminergic fibers examined were small diameter, unmyelinated and mainly restricted to the dorsolateral quadrant on the mfb's anterior–posterior (AP) axis. Fiber densities were similar, except at segment AP −2.8 mm: here FSL males had significantly fewer DA fibers vs. Control males, and vs. FSL females. Divergence in phenotype and stimulation‐evoked responses reported previously could be due to differences in the recruited DAergic fibers.

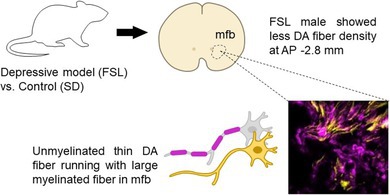

**Summary**

- Myriad factors influence the mechanisms of action of mfb DBS. The study investigated the trajectory, density and myelination of mesocorticolimbic DAergic fibers coursing through the mfb in FSL/Control, male/female animals.
- Fibers in both groups were small diameter, unmyelinated, localized in the mfb's dorsolateral quadrant.
- Fiber density along the mfb axis was comparable, the exception being segment AP −2.8 mm: here FSL males had significantly fewer DA fibers vs. SD males, and vs. FSL females. This area corresponds to the preclinical mfb DBS target.
- Previously reported divergence in phenotype and stimulation‐evoked responses could be due to differences in the recruited DAergic fibers.

## 1 Introduction

Major Depressive Disorder (MDD) is a complex neuropsychiatric disorder affecting approximately 300 million of the global population (Herrman et al. 2022). Although most individuals with MDD respond to conventional treatments, up to a third of the patients remain refractory and are categorized as having treatment‐resistant depression (TRD). Open‐label clinical trials have shown that Deep Brain Stimulation (DBS) of the superolateral branch of the Medial Forebrain Bundle (“MFB” is used to refer to human and “mfb” to rodent) can rapidly and sustainably relieve depressive symptoms in TRD (Coenen et al. 2022, 2019; Fenoy et al. 2022, 2018; Schlaepfer et al. 2013). Experimental mfb DBS can also induce antidepressant effects in animal models (Döbrössy et al. 2021; Furlanetti, Döbrössy, et al. 2015; Thiele et al. 2018). The mechanisms of action of mfb DBS are likely to be complex and influenced by a myriad of factors. Dopaminergic (DAergic) neurons, primarily originating from the ventral tegmental area (VTA) project to key areas associated with the neurocircuitry of MDD such as the nucleus accumbens (NAc) and the prefrontal cortex (PFC) (Devoto et al. 2019; Döbrössy et al. 2021; Russo and Nestler 2013), and play a role in the modulation of mood, motivation, and reward mechanisms (Döbrössy et al. 2021). The selective optogenetic activation of DAergic fibers can alleviate depressive and anxiety‐like symptoms in chronic mild stress animal models (Tong et al. 2022; Tye et al. 2013).

Experimental research has demonstrated that mfb DBS in rodent models can evoke DA release in the NAc and PFC, proposing a mechanism by which stimulation could acutely and chronically modulate behavior (Ashouri Vajari et al. 2020; Dandekar et al. 2017; Miguel Telega et al. 2025, 2022). Studies have also probed into how varying DBS parameters affect DA neuronal firing and have shown that differing DBS parameters can result in varied levels of DA release in the NAc in the Flinders Sensitive Line (FSL) rodent depression model, and controls (Ashouri Vajari et al. 2020; Miguel Telega et al. 2025, 2022). The activation of neurons through axon stimulation depends on the injected current density which in turn is influenced by the electrode‐to‐axon distance and the intrinsic properties of the targeted neural fibers. Myelinated and large diameter fibers typically possess superior chronaxie and excitability characteristics and can be activated by a millisecond pulse width stimulation (Ranck, Ranck Jr 1975). In contrast, unmyelinated fibers require microsecond pulse width stimulation to be activated (Tehovnik et al. 2006). Consequently, understanding the spatial distribution and myelination profiles of DAergic fibers within the mfb is crucial for elucidating the underlying mechanisms and enhancing the effectiveness of DBS.

The current study investigated and compared the trajectory via the mfb, the density and the myelination of the mesocorticolimbic DAergic projections in the depressive model and the control groups, and across the two sexes. The segments selected for analysis along the anterior–posterior axis of the mfb correspond to segments described in the Paxinos and Watson rat brain atlas (Paxinos and Watson 2013). By analyzing and better understanding these anatomical and morphological features, the study aims to shed light on the mechanisms by which mfb DBS influences DA neuron activity in healthy and pathological states.

## 2 Methods and Materials

### 2.1 Animals

Twelve adult rats aged between 10 and 12 weeks, weighing between 270 and 540 g, were used in the study: six FSL (rodent depression model; three males/three females) and six Sprague–Dawleys (SD, controls; three males/three females). Animals had *ad libitum* access to food and water and were housed three rats/cage at the Neurozentrum Freiburg animal facility. Classic housing conditions with a 12‐h light/dark cycle were employed to ensure a stable circadian rhythm for the animals. All experimental procedures were conducted in strict adherence to the guidelines set by the Regierungspräsidium Freiburg (TVA G20‐97) and adhered to the Animals (Scientific Procedures) Act 1986, International Association for Study of Pain (Zimmermann 1983) and ARRIVE guidelines (Kilkenny et al. 2010).

### 2.2 Immunohistochemistry

Rats were administered a terminal dose of ketamine (400 μL of 100 mg/mL) combined with xylazine (200 μL of 20 mg/mL), and intracardially perfused with ice‐cold 0.3% phosphate‐buffered saline (PBS) followed by 4% paraformaldehyde (PFA) in 0.1 M PBS at pH 7.4. After perfusion, the extracted brains were immersed in a 30% sucrose solution at 4°C until fully submerged, and coronally cut into 40 μm slices. Two staining protocols were used: First, to permit the assessment of the localization and the density of the DAergic neurones traversing the mfb; and second, to study the myelination state of the DAergic projections in the mfb. Localization/density: 1 in 6 coronal sections (240 μm apart) were selected and rinsed three times for 10 min in 0.3% PBS. Next, sections were placed in blocking solution containing 5% BSA and 0.3% Triton X‐100 in PBS for an hour to prevent non‐specific binding. The sections were incubated simultaneously overnight with rabbit anti‐Tyrosine Hydroxylase (TH, 1:200, Merck, AB152) and mouse anti‐Dopamine‐β‐Hydroxylase (DβH, 1:700, Chemicon, MAB308) primary antibodies to assess DA and noradrenaline fiber distribution. After three PBS washes, sections were simultaneously treated with secondary antibodies goat anti‐rabbit 568 (1:500 for myelin, Life technology, A11011), and goat anti‐mouse 488 (1:500, Life technology, A11001) for 5 h. A subsequent triple wash in 0.3% PBS was performed before mounting the sections, sealing them with mounting medium, and left to dry in the dark.

The myelin sheaths staining used an 8‐day on‐slide protocol. Post a triple PBS wash, sections were treated with 0.5% NaBH4 for 30 min, followed by antigen retrieval in citrate buffer (pH 6.0) at 50°C for 20 min. After four 5‐min PBS washes, sections were blocked and incubated simultaneously with primary rabbit anti‐myelin (1:100, Abcam, ab216590) and mouse anti‐TH antibodies (1:2000, Sigma, T1299) for 5 days at 4°C. After washing, sections were exposed simultaneously to secondary anti‐rabbit 568 (1:500, Thermo Fisher, Alexa Fluor 568, A11011) and anti‐mouse 488 antibodies (1:500, Thermo Fisher, Alexa Fluor 488, A11001) for 24 h. The following day, sections were treated with DAPI (1:1000, Sigma, D9542) for 20 min in the dark at room temperature, washed, and left to dry overnight. The sections were dehydrated with 70% ethanol, treated with an autofluorescence elimination reagent (Millipore, # 2160), sealed with mounting medium and kept away from light.

Epifluorescence visualization used Axioscan 7 (Zeiss, Germany). The acquired images were brightness and contrast adjusted and exported by ZEN 2.5 software. A confocal image was captured using a Nikon Ti Eclipse microscope equipped with a C2 point‐scanning confocal unit, a 100× objective lens (NA 1.45), appropriate lasers (408, 488 and 561 nm) and emission filters (460/50, 515/30, 600/50 nm). The pinhole size was set to 30 μm, pixel size to 90 nm, and Z‐slice interval to 0.12 μm.

### 2.3 Fiber Quantification

**FIGURE 1:**
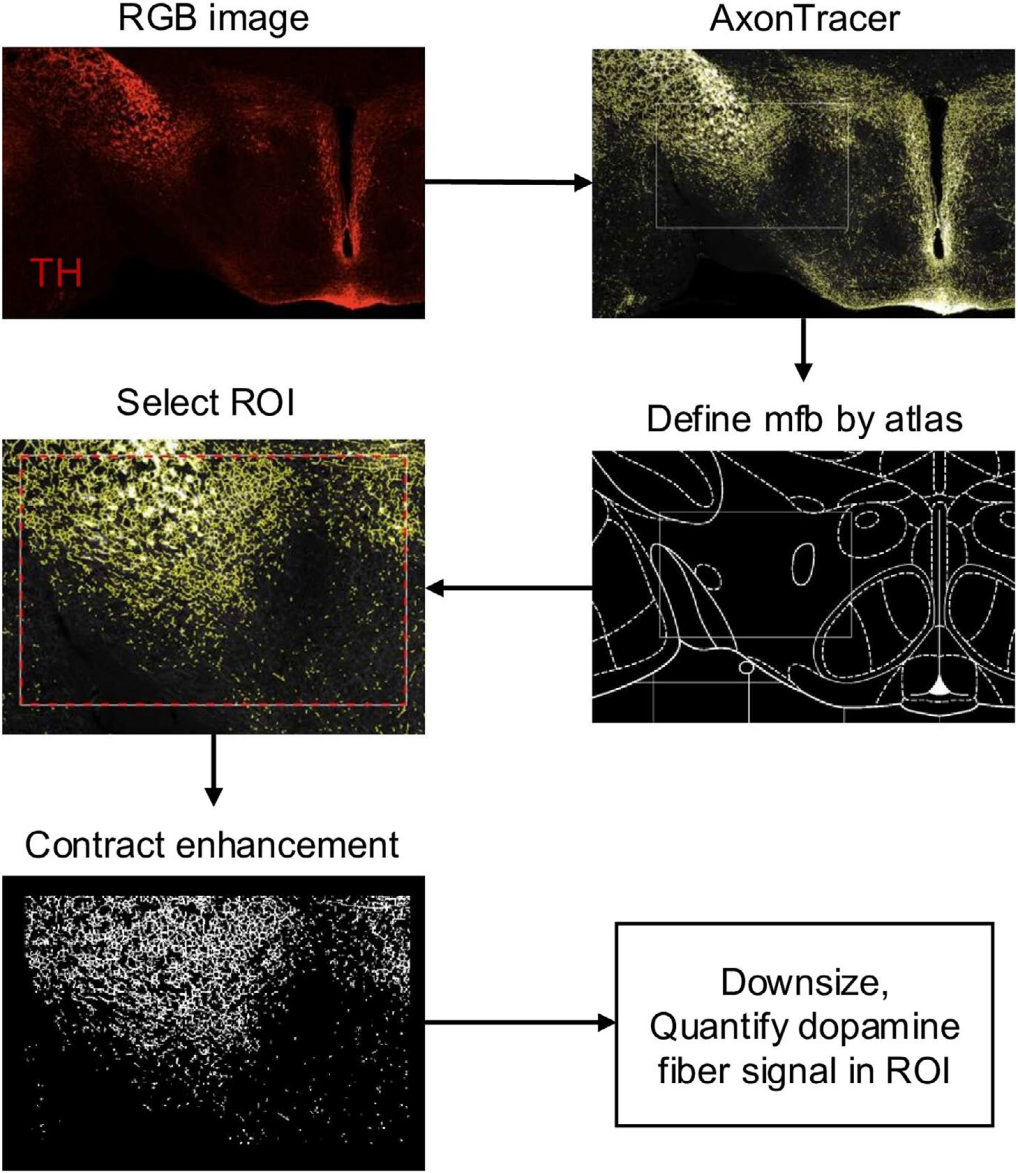
Fiber quantification process. The images summarize the step‐by‐step process used to quantify the DAergic (TH+/DßH−) fibers traversing the mfb. See the Section 2 for a more detailed description.

The methodology used is graphically summarized in Figure 1. Exported images were aligned to anatomical structures based on the atlas “The Rat Brain in Stereotaxic Coordinates – 7th edition” (Paxinos and Watson 2013). Six segments along the mfb's anterior–posterior (AP) axis (based on Nieuwenhuys' anatomical description of the rodent mfb; Nieuwenhuys et al. 1982) were used for the analysis: AP −1.1 mm, −1.4, −2.0, −2.8, −3.7, and −4.4 mm. The images were aligned with the identical sized atlas template (750 × 950 pixels) by Photoshop (Adobe System). Positive stained axons were identified by the ImageJ plugin, AxonTracer (Patel et al. 2018). The parameter applied for the axon detection sensibility threshold was from 6 to 30—based on the fluorescent intensity of the RGB image and minimal axon length 1 pixel—converting the positive staining into unique yellow pixels (0, 0, 255). The yellow pixels were downsized by 0.5 and recognized by MATLAB. The yellow pixels represented non‐zero elements were saved. “Non‐zero elements” are taken as a direct index of DA fiber signal in the region of interest. The DAergic fibers were identified as fibers that were TH+/DβH−. For the distribution of DAergic fibers, the non‐zero elements of TH staining were subtracted from the non‐zero elements of DβH staining to obtain the non‐zero elements representing DAergic fibers. The DAergic fibers heat maps were superimposed on the blank template. The percentage of non‐zero elements in the 750 × 950 pixels atlas was compared between different groups.

### 2.4 Statistics Analysis

All data analyses were performed using GraphPad Prism. A two‐way ANOVA was used to assess the interaction between animal strains and sex. When a significant interaction was observed, post hoc comparisons were conducted using the Bonferroni test with adjusted *p*‐values. Graphs and corresponding statistical outputs were generated in GraphPad Prism. A *p*‐value of < 0.05 was considered statistically significant. All the statistical calculations are now summarized in Tables S1 and S2.

## 3 Results

### 3.1 Spatial Localization and DAergic Fiber Density Passing Through the Mfb

**FIGURE 2:**
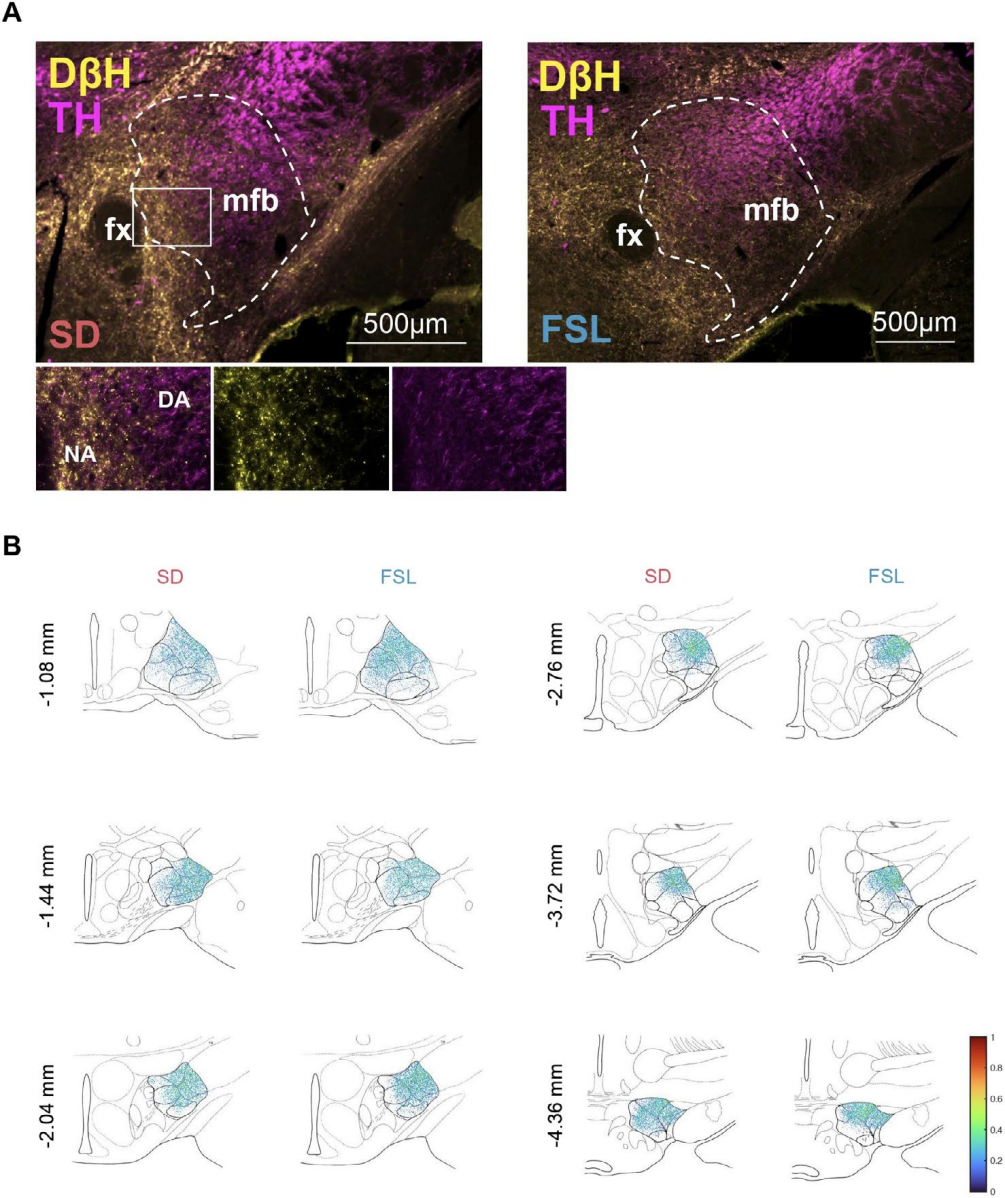
(A, B) Visualization of DAergic Fibers in Rat Brain. (A) Representative fluorescence images show the distribution of DAergic fibers and noradrenergic fibers in the medial forebrain bundle (mfb) of SD and depressive model FSL rats at the AP level−2.8 mm, the common site for stimulation electrode implantation. (B) Composite heat maps illustrating the DAergic fiber density across six AP levels (−1.1, −1.4, −2.0, −2.8, −3.7, and −4.4 mm from bregma) based on quantifications from six animals per group (SD and FSL).

The study analyzed the distribution and the position of the DA fibers traversing the rodent mfb in the two experimental groups, and in both males and females. A distinctive spatial organization and segregation between the DAergic and noradrenergic fibers was noted, with the two converging only within a transitional zone (Figure 2A). Histological examination of brain sections at AP levels −1.1, −1.4, −2.0, −2.8, −3.7, and −4.4 mm, showed DA fibers to project as clustered neuronal bundles primarily within the dorsolateral quadrant of the mfb in both SD and FSL rats (Figure 2B).

**FIGURE 3:**
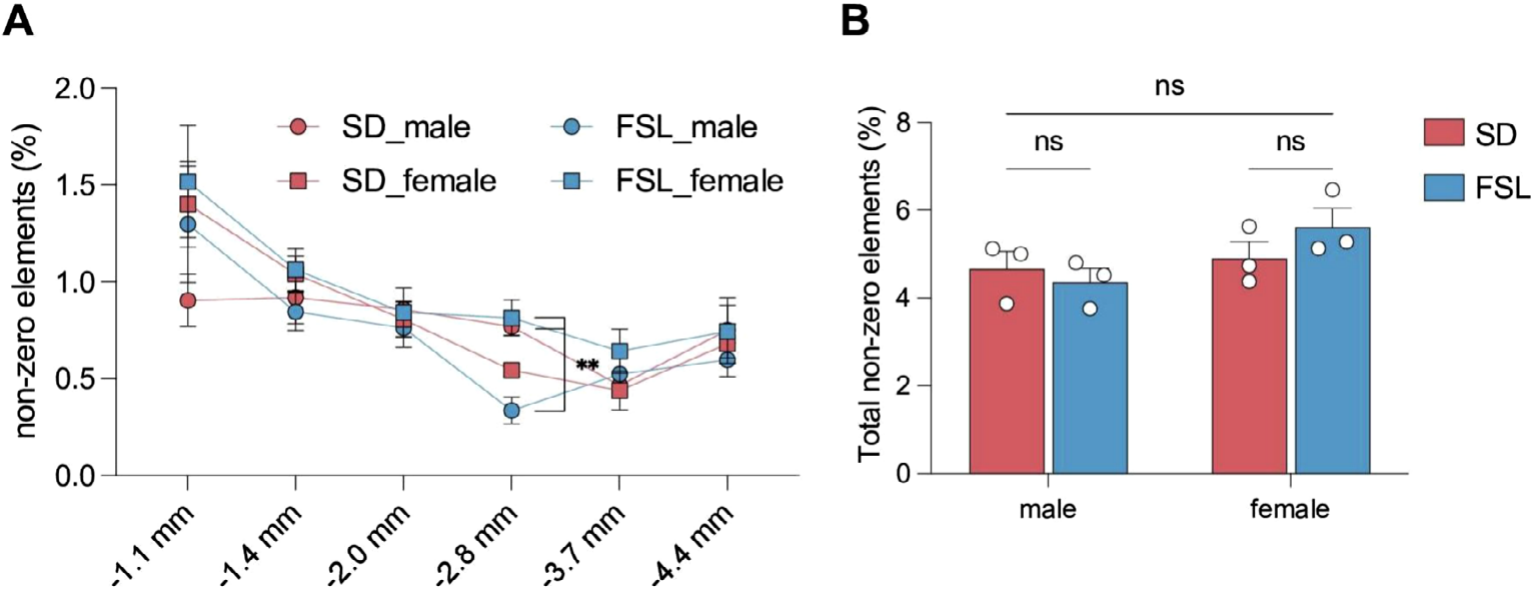
(A, B) Quantitative Analysis of DAergic Fiber Density. (A) Measurement of DAergic fiber density shown as the percentage of traced yellow non‐zero pixels within a defined 750 × 950 pixel atlas region across six anteroposterior (AP) levels: −1.1, −1.4, −2.0, −2.8, −3.7, and −4.4 mm from bregma. Data are represented for both sexes in a depression model (FSL) and the control group (SD). (B) Sex‐based comparison of DAergic fiber density in the depression model and control groups. No statistically significant differences were found between males and females within each group, nor between the depression model and control groups at the significance level of *p* < 0.05.

Investigating differences in fiber density, a two‐way ANOVA demonstrated a significant interaction between the experimental groups and sexes at AP −2.8 mm (Figure 3A; *F*_(1,8)_ = 30.14, *p* = 0.0006). Bonferroni's post hoc test revealed a significant reduction in DA fibers amongst the male FSLs compared to male SDs (*p* = 0.0084), as well as significantly fewer fibers amongst the male FSLs compared to female FSLs (*p* = 0.0045). These observations were specific to segment AP −2.8 mm, and no significant differences in the total density of DA fibers were seen between the two experimental groups or across the sexes (Figure 3B; *F*_(1,8)_ = 1.802, *p* = 0.2163). The data points towards a structural difference—specific to this segment on the mfb—in the DAergic fibers between FSL and SD males, and across the sexes within the FSL animals.

### 3.2 Myelination of the DAergic Fibers Passing Through the Mfb

The myelination state of the DAergic fibers passing through the dorsolateral quadrant of the mfb in FSL and SD animals was assessed. Given that electrical stimulation preferentially activates large myelinated fibers with the lowest chronaxie, differences in the myelination profile of these fibers can influence the functional dynamics of DBS.

**FIGURE 4:**
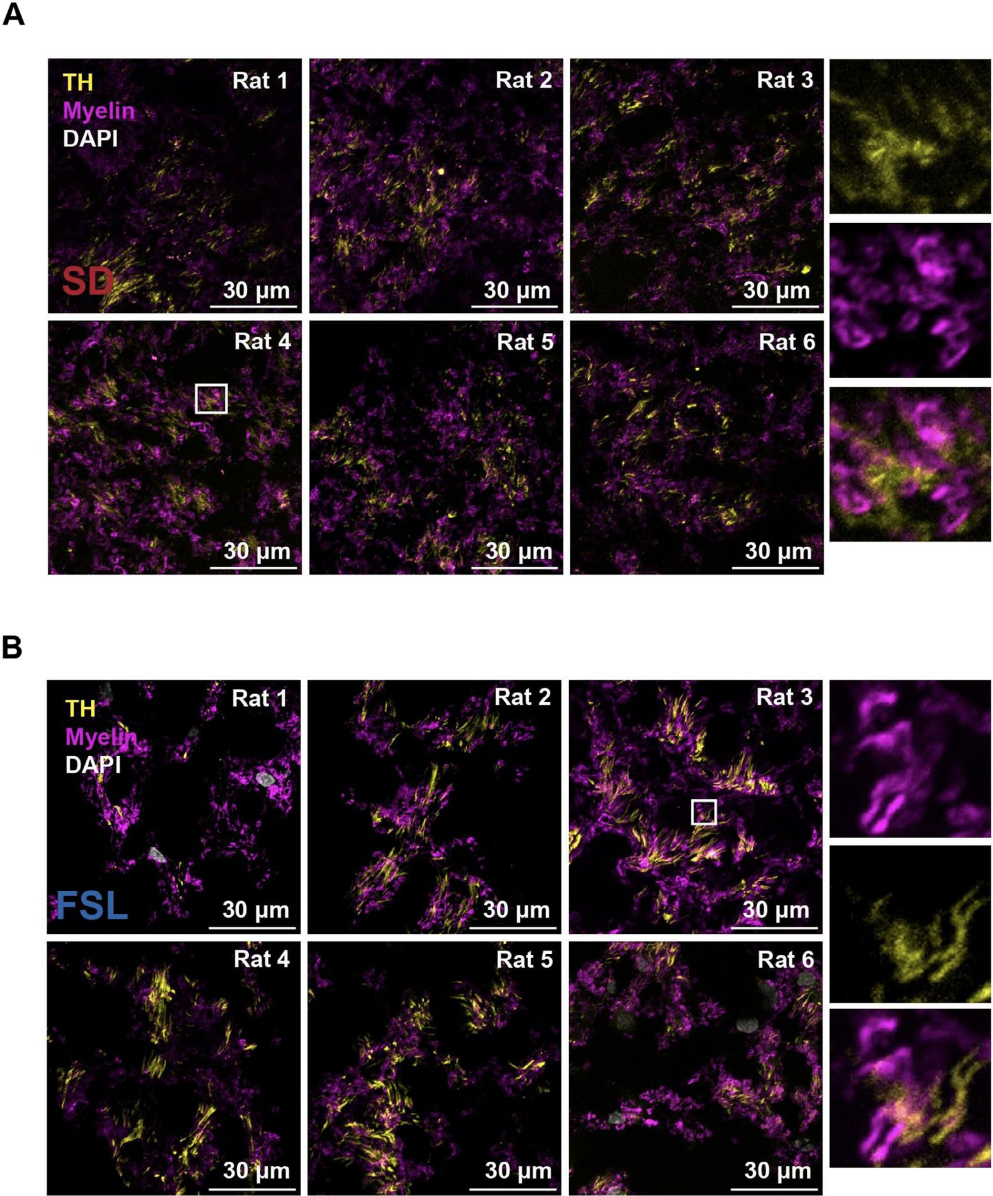
(A, B) Immunofluorescent Labeling of DAergic Fibers and Myelin in SD and FSL Rat Brains. (A) Series of fluorescence micrographs showing the distribution of DAergic fibers (TH; yellow) and myelin (magenta) in the medial forebrain bundle (mfb) of SD rats. Each panel represents an individual rat (SD 1 to SD 6), with cell nuclei counterstained using DAPI (white). Insets provide a higher magnification to emphasize the TH‐positive fibers projecting along with the large myelinated neuron in mfb. (B) Corresponding fluorescence images for FSL rats, depicting similar distributions of TH‐positive DAergic fibers and myelin in mfb. Insets magnify the area within the white square, illustrating the detailed architecture and interaction of TH‐positive fibers with large myelinated fibers.

Myelination was investigated exclusively at the mfb segment (AP −2.8 mm) where fiber density differences across experimental groups were identified. Segment AP −2.8 mm corresponds to the implantation site of the DBS electrode during preclinical studies. Most of the fibers were spatially distinct from the DβH+ NA fibers which were located medially and consistently contained bulged varicosities (Figure 2A). Under confocal microscopy at 100× magnification, medium and large myelinated fibers were noted to course in the proximity of the DA fibers. Mixing with myelinated fibers in mfb, the DA fibers, identified by TH staining, did not co‐localize with myelin neither in the SD nor in the FSL groups. No wrapping or sheathing of myelin was visualized around the TH+ structures (Figure 4A,B).

## 4 Discussion

Major Depressive Disorder patients refractory to conventional treatments have responded well to Medial Forebrain Bundle (“MFB” in humans, “mfb” in rodents) DBS in a series of clinical trials carried out to date (Coenen et al. 2022, 2019; Fenoy et al. 2022, 2018; Schlaepfer et al. 2013). The concentration of the fibers coursing the MFB makes it a singularly strategic target for DBS because the stimulation of a single area can regulate—directly or indirectly—neuronal activity on multiple distal functional networks including the Affective, Default Mode, Reward and Cognitive Control networks (Hitti et al. 2020; Johnson et al. 2024). One hypothesis is that the observed antidepressant effects are—in part—based on the modulation of the mesocorticolimbic DAergic pathways (Döbrössy et al. 2021; Pallikaras and Shizgal 2022a). DA transmission, via neurones originating in the VTA and projecting to NAc/mPFC through the mfb, significantly contributes to mood regulation, motivation, reward‐oriented behaviors, and has been implicated in the neurocircuitry of depression pathology (Klein et al. 2019). In order to shed light on the antidepressant mechanisms of action of mfb DBS, the current anatomical study used the Flinders Sensitive Line (FSL) rodent depression model and healthy controls to map out the distribution/spatial location and the myelination state of the midbrain DAergic fibers passing through the mfb.

Our understanding today of the anatomy of the rodent mfb is based on a series of classical papers by Nieuwenhuys and colleagues (Geeraedts et al. 1990a, 1990b; Nieuwenhuys et al. 1982; Veening et al. 1982). The mfb is a fascicle of fibers of diverse neurotransmitter and modulator systems that bi‐directionally connect mid/hindbrain with multiple forebrain regions. The ascending and descending fiber bundles are densely packed as they pass through the anterior–posterior axis of the mfb/MFB. In a previous work we used whole brain, monosynaptic viral tracing mapping of the VTA ascending projecting neurons in FSL and Control animals (Tong et al. 2024). The data showed the first characterization of inputs to different VTA ascending projection neurons, and it identified numerous critical differences in the connectome of the midbrain–forebrain system in the FSL model. Based on the images available from the Paxinos and Watson rat brain atlas, the current study selected a series of six coronal segments (AP −1.1, −1.4, −2.0, −2.8, −3.7, and −4.4 mm) along the rodent's mfb axis to analyze. Data confirm that—independent of the experimental group—DAergic projections were similarly small diameter, and the clustered neuronal bundles were preferentially localized in the dorsolateral quadrant of the mfb. Fiber density, an index of the fiber numbers, was also overall indistinguishable across the FSLs and Controls, with the exception at segment AP −2.8 mm. At this area, we observed a significant reduction in DAergic fibers selectively amongst the male FSL animals in comparison to both the FSL females and the Control males.

Sparser DAergic fibers in the FSLs at around AP −2.8 mm, but not at any of the other segments either more anterior or posterior, are intriguing for at least two reasons.

Firstly, the area at AP −2.8 mm corresponds approximately to the position of the lateral hypothalamus, and the reduced DAergic fibers specifically at this area could indicate reduced DAergic input into this structure. Orexinergic neurones from the lateral hypothalamus project to DA neurons in the VTA and can modulate DAergic transmission going through the mfb via the mesocorticolimbic pathways. The consequence of reduced DAergic fiber density in the FSL males could mean less optimal reciprocal connections and regulation between the dopaminergic and orexinergic systems, and could impact motivation and reward‐oriented behaviors (Harada et al. 2024; Vaseghi et al. 2022). FSL animals spontaneously show a “depressive‐like” phenotype including increased immobility on the Forced Swim Test, increased anxiety, reduced motivation, deficits in cognitive, learning and memory tasks, and reduced body weight (Cook et al. 2017; Overstreet and Wegener 2013; Steyn 2025; Thiele et al. 2016), and a stronger phenotype has been observed and previously reported in FSL males compared to FSL females (Thiele et al. 2016). The clinical reality is that women, compared to men, are almost twice as likely to suffer from major depressive disorder (Noble 2005; Salk et al. 2017), as well as from treatment‐resistant depression (TRD) (Liu et al. 2021). However, the clinical manifestations and assessment of depression are more nuanced compared to the broad‐brush, “depressive‐like” phenotype defined in experimental research which is useful, albeit a poor replication of the human predicament (Becker et al. 2021; Planchez et al. 2019).

Secondly, the area at AP −2.8 mm corresponds to the position in the mfb where we typically place the DBS electrodes to stimulate the experimental animals. The original choice for the DBS electrode placement was based on our and others' data (Ikemoto 2010, 2007) that at this anterior–posterior (−2.8 mm) and medial‐lateral (±1.7) space in the mfb, the mesocortical DAergic fibers were predominantly separated away from the nigrostriatal DAergic fibers and thus could be more selectively targeted even with electrical stimulation.

The current study was exclusively anatomical, but earlier work from our group and others confirms that mfb DBS in FSL animals can reverse a variety of behavioral symptoms, including cognitive, learning/memory tasks (Thiele et al. 2018, 2016), and improve stress coping behaviors (Döbrössy et al. 2015; Furlanetti, Coenen, et al. 2015; Furlanetti, Döbrössy, et al. 2015). The differences in the DAergic fiber density at the level of the lateral hypothalamus in the mfb imply differences in connectivity that could partially explain both behavioral (Cook et al. 2017; Thiele et al. 2018, 2016) and diverging accumbal DA release patterns evoked by mfb DBS (Ashouri Vajari et al. 2020; Miguel Telega et al. 2025). MRI‐based studies in clinically depressed patients have shown both anatomic/connectomic and symptomatic/phenotypic sexual dimorphisms, implicating differences in temporal lobe components and limbic pathways, or more anger attacks/aggression/substance use in men and more appetite disturbance/impaired sleep/depressed mood in women (Cavanagh et al. 2017, 2016; Mohammadi et al. 2023).

Interestingly, sexual dimorphisms are also present in the response to DBS: limited data from small sample‐sized open‐label clinical trials indicate that females with TRD respond at higher rates to mfb DBS treatment than males, and this is the case also with most other DBS targets (Patel et al. 2024).

High frequency electrical stimulation is non‐selective with respect to neuronal phenotype, which implies that mfb DBS probably achieves its physiological/behavioral effects by acting on multiple neurotransmitter systems directly in the vicinity of the electrical field and indirectly via modulating distal networks. We have shown using multiple neurotransmitter monitoring techniques that mfb DBS induces acute and enduring secretion of DA and noradrenaline in forebrain structures such as the mPFC and NAc, and the release patterns are different in the FSL and control animals (Ashouri Vajari et al. 2020; Miguel Telega et al. 2025, 2022). Beyond monoaminergic modulation, mfb DBS also affects additional complex processes involving GABAergic inhibitory control of neural excitability and network synchrony (Bühning et al. 2022; Duan et al. 2025). Different biophysical properties of the axons and neuronal cell bodies—that constitute any given area—and the specific DBS stimulation parameters applied, will collectively determine which neuronal components—and in what sequence—will be preferentially stimulated or modulated (Ramasubbu et al. 2018). Assessing the state of myelination of the fibers traversing the DBS target area can significantly enhance our understanding of the proximal and distal mechanisms of action of electrical stimulation (Anderson et al. 2020). Myelin decreases axonal capacitance, reducing the energy requirements for action potential propagation; conversely, activating unmyelinated axons requires higher charge density (Suminaite et al. 2019).

The current investigation confirmed the unmyelinated state of the mesocorticolimbic DAergic fibers passing through the mfb, and this was the case in both the FSL and the Control animals. In both experimental groups, the small diameter unmyelinated DAergic fibers were found to course alongside medium and large myelinated axons, as evidenced by the fluorescence micrographs. These myelinated axons are likely descending, corticofugal, glutamatergic fibers, projecting via the mfb to midbrain, brain stem and spinal cord areas (Usrey and Sherman 2019). Considering that extracellular electrical stimulation selectively activates fibers with the lowest threshold for activation, it is likely that the typical stimulation parameters used in our preclinical studies (130 Hz, 100 μs pulse‐widths, 300 μA) will preferentially—and directly—activate the larger myelinated fibers. The thin, unmyelinated DAergic—but also serotonergic and noradrenergic—fibers originating from mid and hindbrain nuclei and projecting onto multiple forebrain and cortical structures, require higher current density to be activated. The mfb stimulation‐evoked antidepressant effects described in the experimental and clinical literature, are likely to be the sum of the cascade of both direct (non‐monoaminergic) and indirect (monoaminergic) activation of the neurotransmitter and neuromodulator systems traversing the mfb (Pallikaras and Shizgal 2022a, 2022b; Yeomans 1989).

The study has a number of limitations. Only anatomical methods were used and animals did not receive mfb DBS. Structure and function speculations could only be done in the context of previous studies with integrated stimulation and behavioral analysis. The experimental group sizes were moderate and contained both male and female rats. Finally, there are limitations in extrapolating findings from rodent to human anatomy. In primates, and especially in humans, the connectivity and myelination patterns are significantly more complex and less studied (Coenen et al. 2018, 2022, 2023).

The emerging data provide information about the spatial distribution and myelination status of DAergic fibers coursing through a large segment of the rodent mfb, and compare these features across the FSL model and controls. Special attention was paid to the DBS target area used in preclinical stimulation experiments. In conclusion, the DAergic fibers traversing the anterior–posterior axis of the mfb in both FSL and control animals were small diameter, unmyelinated and mainly restricted to the dorsolateral quadrant. DAergic fiber density along the assessed mfb segments was indistinguishable across the experimental groups, with the exception at the level of the lateral hypothalamus, where fewer fibers were detected in the male FSLs compared to female FSLs and male controls. This segment is particularly relevant as it corresponds to the target for mfb DBS in preclinical models. Reduced connectivity between the VTA and the lateral hypothalamus probably contributes to the model's “depressive‐like” phenotype, and the differences observed in mfb DBS evoked DA release as reported by our group previously (Ashouri Vajari et al. 2020; Miguel Telega et al. 2025, 2022). Future studies, integrating anatomical, physiological and behavioral methods can build on these data to shed further light on the mechanisms of mfb DBS in models of psychiatric disorders and in its clinical application.

## Author Contributions

Zhuo Duan, Yixin Tong and Máté D. Döbrössy conceived the study; Zhuo Duan performed the experiments; Zhuo Duan, Xiongpeng Weng and Yixin Tong analyzed the data; Zhuo Duan, Yixin Tong and Máté D. Döbrössy interpreted the data; Zhuo Duan, Máté D. Döbrössy and Volker A. Coenen wrote the manuscript; Máté D. Döbrössy supervised the project; all authors read and approved the manuscript.

## Ethics Statement

All experimental procedures were conducted in strict adherence to the guidelines set by the local Ethics Committee, the Regierungspräsidium Freiburg, Germany. The Ethics Approval number is TVA G20‐97.

## Conflicts of Interest

The authors declare no conflicts of interest.

## Supporting information


**Table S1:** Detail summary of all statistical analyses.
**Table S2:** Bonferroni‐corrected post hoc comparisons at −2.8 mm Bregma (Figure 1).

## Data Availability

The data are available on request from the corresponding author.
