# Supplementary material for: Fiber Distribution and Myelination of Dopaminergic Neurons in the Medial Forebrain Bundle of a Rodent Depression Model
Source: J Neurosci Res. 2025 Nov 16;103(11):e70093. doi: 10.1002/jnr.70093 (PMC12619984; doi:10.1002/jnr.70093)
Supplement: Supplementary file 1 — Table S1: Detail summary of all statistical analyses. Table S2: Bonferroni‐corrected post hoc comparisons at −2.8 mm Bregma (Figure 1). [file JNR-103-e70093-s001.docx]

| **Table S1 Statistical analysis** | | | | | | | | | | |
| --- | --- | --- | --- | --- | --- | --- | --- | --- | --- | --- |
| **Figure** | **Subject** | **Group** | | **Mean** | **SEM** | **n** | **Method** | **Value** | | **p** |
| Fig. 1 | -1.1 mm | SD | male | 0.90 | 0.14 | 3 | two-way ANOVA | animal strain x sex | F (1, 8) = 0.3148 | P=0.5901 |
|  |  |  | female | 1.40 | 0.22 |  |  | animal strain | F (1, 8) = 2.134 | P=0.1822 |
|  |  | FSL | male | 1.30 | 0.30 | 3 |  | sex | F (1, 8) = 1.075 | P=0.3302 |
|  |  |  | female | 1.52 | 0.29 |  |  |  |  |  |
| Fig. 1 | -1.4 mm | SD | male | 0.92 | 0.14 | 3 | two-way ANOVA | animal strain x sex | F (1, 8) = 0.1878 | P=0.6762 |
|  |  |  | female | 1.04 | 0.09 |  |  | animal strain | F (1, 8) = 2.411 | P=0.1591 |
|  |  | FSL | male | 0.85 | 0.10 | 3 |  | sex | F (1, 8) = 0.05819 | P=0.8154 |
|  |  |  | female | 1.06 | 0.11 |  |  |  |  |  |
| Fig. 1 | -2.0 mm | SD | male | 0.86 | 0.04 | 3 | two-way ANOVA | animal strain x sex | F (1, 8) = 0.4991 | P=0.5000 |
|  |  |  | female | 0.81 | 0.09 |  |  | animal strain | F (1, 8) = 0.02045 | P=0.8898 |
|  |  | FSL | male | 0.76 | 0.10 | 3 |  | sex | F (1, 8) = 0.1001 | P=0.7598 |
|  |  |  | female | 0.84 | 0.12 |  |  |  |  |  |
| Fig. 1 | -2.8 mm | SD | male | 0.77 | 0.04 | 3 | two-way ANOVA | animal strain x sex | F (1, 8) = 30.14 | P=0.0006 |
|  |  |  | female | 0.54 | 0.03 |  |  | animal strain | F (1, 8) = 3.930 | P=0.0827 |
|  |  | FSL | male | 0.34 | 0.07 | 3 |  | sex | F (1, 8) = 1.599 | P=0.2417 |
|  |  |  | female | 0.82 | 0.09 |  |  |  |  |  |
| Fig. 1 | -3.7 mm | SD | male | 0.47 | 0.04 | 3 | two-way ANOVA | animal strain x sex | F (1, 8) = 0.5948 | P=0.4627 |
|  |  |  | female | 0.44 | 0.10 |  |  | animal strain | F (1, 8) = 0.2440 | P=0.6346 |
|  |  | FSL | male | 0.52 | 0.10 | 3 |  | sex | F (1, 8) = 1.941 | P=0.2011 |
|  |  |  | female | 0.64 | 0.11 |  |  |  |  |  |
| Fig. 1 | -4.4 mm | SD | male | 0.75 | 0.17 | 3 | two-way ANOVA | animal strain x sex | F (1, 8) = 0.7241 | P=0.4196 |
|  |  |  | female | 0.68 | 0.10 |  |  | animal strain | F (1, 8) = 0.08909 | P=0.7729 |
|  |  | FSL | male | 0.60 | 0.09 | 3 |  | sex | F (1, 8) = 0.1231 | P=0.7348 |
|  |  |  | female | 0.74 | 0.14 |  |  |  |  |  |
| Fig. 2 | Total fibers | SD | male | 4.67 | 0.40 | 3 | two-way ANOVA | animal strain x sex | F (1, 8) = 1.802 | P=0.2163 |
|  |  |  | female | 4.91 | 0.37 |  |  | animal strain | F (1, 8) = 0.2899 | P=0.6050 |
|  |  | FSL | male | 4.36 | 0.31 | 3 |  | sex | F (1, 8) = 3.965 | P=0.0816 |
|  |  |  | female | 5.62 | 0.42 |  |  |  |  |  |

| **Table S2 Post-hoc analysis (Bonferroni test)** | | | | | | |  |
| --- | --- | --- | --- | --- | --- | --- | --- |
| **Subject** | **p** | | | | | | |
| -2.8 |  |  | SD | | FSL | | |
|  |  |  | male | female | male | female | |
|  | SD | male |  |  |  |  | |
|  |  | female | 0.2287 |  |  |  | |
|  | FSL | male | 0.0084 | 0.3048 |  |  | |
|  |  | female | >0.9999 | 0.1044 | 0.0045 |  | |
